# VPAC1 receptor (Vipr1)-deficient mice exhibit ameliorated experimental autoimmune encephalomyelitis, with specific deficits in the effector stage

**DOI:** 10.1186/s12974-016-0626-3

**Published:** 2016-06-29

**Authors:** Catalina Abad, Bhavaani Jayaram, Laurine Becquet, Yuki Wang, M Sue O’Dorisio, James A. Waschek, Yossan-Var Tan

**Affiliations:** Department of Psychiatry, David Geffen School of Medicine, University of California, Los Angeles, USA; Inserm U905, Institute for Research and Innovation in Biomedicine (IRIB), University of Rouen, Normandy, France; Department of Pediatrics and Holden Comprehensive Cancer Center, RJ and LA Carver College of Medicine, University of Iowa, Iowa City, 52242 IA USA

**Keywords:** Multiple sclerosis, Experimental autoimmune encephalomyelitis, Neuropeptide, VPAC1, Neuroimmunomodulation

## Abstract

**Background:**

Vasoactive intestinal peptide (VIP) and pituitary adenylyl cyclase-activating polypeptide (PACAP) are two highly homologous neuropeptides. In vitro and ex vivo experiments repeatedly demonstrate that these peptides exert pronounced immunomodulatory (primarily anti-inflammatory) actions which are mediated by common VPAC1 and VPAC2 G protein-coupled receptors. In agreement, we have shown that mice deficient in PACAP ligand or VPAC2 receptors exhibit exacerbated experimental autoimmune encephalomyelitis (EAE). However, we observed that VIP-deficient mice are unexpectedly resistant to EAE, suggesting a requirement for this peptide at some stage of disease development. Here, we investigated the involvement of VPAC1 in the development of EAE using a VPAC1-deficient mouse model.

**Methods:**

EAE was induced in wild-type (WT) and VPAC1 knockout (KO) mice using myelin oligodendrocyte glycoprotein 35–55 (MOG_35–55_), and clinical scores were assessed continuously over 30 days. Immune responses in the spinal cords were determined by histology, real-time PCR and immunofluorescence, and in the draining lymph nodes by antigen-recall assays. The contribution of VPAC1 expression in the immune system to the development of EAE was evaluated by means of adoptive transfer and bone marrow chimera experiments. In other experiments, VPAC1 receptor analogs were given to WT mice.

**Results:**

MOG_35–55_-induced EAE was ameliorated in VPAC1 KO mice compared to WT mice. The EAE-resistant phenotype of VPAC1 KO mice correlated with reduced central nervous system (CNS) histopathology and cytokine expression in the spinal cord. The immunization phase of EAE appeared to be unimpaired because lymph node cells from EAE-induced VPAC1 KO mice stimulated in vitro with MOG exhibited robust proliferative and Th1/Th17 responses. Moreover, lymph node and spleen cells from KO mice were fully capable of inducing EAE upon transfer to WT recipients. In contrast, WT cells from MOG-immunized mice did not transfer the disease when administered to VPAC1 KO recipients, implicating a defect in the effector phase of the disease. Bone marrow chimera studies suggested that the resistance of VPAC1-deficient mice was only minimally dependent on the expression of this receptor in the immunogenic/hematopoietic compartment. Consistent with this, impaired spinal cord inductions of several chemokine mRNAs were observed in VPAC1 KO mice. Finally, treatment of WT mice with the VPAC1 receptor antagonist PG97-269 before, but not after, EAE induction mimicked the clinical phenotype of VPAC1 KO mice.

**Conclusions:**

VPAC1 gene loss impairs the development of EAE in part by preventing an upregulation of CNS chemokines and invasion of inflammatory cells into the CNS. Use of VPAC1 antagonists in WT mice prior to EAE induction also support a critical role for VPAC1 signaling for the development of EAE.

**Electronic supplementary material:**

The online version of this article (doi:10.1186/s12974-016-0626-3) contains supplementary material, which is available to authorized users.

## Background

Vasoactive intestinal peptide (VIP) and pituitary adenylate cyclase-activating polypeptide (PACAP) are two highly homologous neuropeptides of the secretin/glucagon superfamily [1, 2]. They act through three class II G protein-coupled receptors (GPCRs) officially named by International Union of Basic and Clinical Pharmacology (IUPHAR) as VPAC1, VPAC2, and PAC1 [3]. Whereas VPAC1 and VPAC2 bind either peptide with equally high affinity, PAC1 is known to be a PACAP-preferring receptor. Signaling through these receptors mainly involves the Gαs protein and leads to activation of adenylyl cyclase and cAMP production [1]. However, they can also increase inositol triphosphate and intracellular calcium levels through the phospholipase C pathway as well as modulate the activity of phospholipase D, tyrosine kinases, and calcium and potassium channels [1].

Multiple studies in the early 1990s reported the expression of VIP, PACAP, and their receptors in the immune system and were followed up by the discovery of their immunomodulatory properties through a direct action on diverse immune cell subtypes such as T lymphocytes, macrophages, and dendritic cells [4]. These cells constitutively express VPAC1. VPAC2 expression, on the other hand, is very low in resting lymphocytes and macrophages but strongly induced in macrophages after exposure to stimuli such as lipopolysaccharide (LPS), the major component of the outer membrane of Gram-negative bacteria, and in lymphocytes activated with an anti-CD3 antibody [5, 6]. In addition, constitutive expression of VPAC2 in dendritic cells and of PAC1 in macrophages has been reported [7, 8].

VIP and PACAP modulate multiple aspects of immunity, and notably, they are potent anti-inflammatory peptides [4]. In this regard, initial in vitro studies described that VIP and PACAP strongly inhibited the production of a wide range of pro-inflammatory cytokines and chemokines by macrophages stimulated with LPS but stimulated the production of anti-inflammatory cytokines such as interleukin (IL)-10 [9–11]. The efficient blockade of the inflammatory response by these peptides appears to be mediated by their inhibitory action on key signaling pathways common to the expression of several cytokines, notably NF-kB and MAPK [12]. Besides VIP and PACAP actions on innate immunity, other in vitro studies demonstrated that they modulate adaptive immune responses by acting on T helper (Th) differentiation [13, 14]. In concurrence with their generally anti-inflammatory actions, it was found that VIP and PACAP inhibit Th1 and potentiate Th2 responses. This was demonstrated to be mediated at least in part by actions on dendritic cell co-stimulatory molecules and cytokine/chemokine production [7, 11, 15–17]. Similarly, human plasmacytoid dendritic cells activated with CpG oligonucleotides stimulate CD4^+^ T cell proliferation and cytokine production ex vivo. The addition of VIP during dendritic cell activation resulted in decreased interferon (IFN)γ production and CD4^+^ T cell proliferation, generating a Th2-like response [18]. Other in vitro and in vivo studies have suggested that VIP and PACAP directly promote specific Th2 cell survival and Th2 memory cell generation [19–21].

More recent studies have demonstrated the ability of these peptides to induce regulatory T cells (Tregs) [22–27]. Due to their anti-inflammatory actions on innate and adaptive immune function, VIP and PACAP administration reduced clinical symptoms and pathological changes in murine models of several inflammatory and Th1-driven autoimmune conditions, including rheumatoid arthritis, Crohn’s disease, and multiple sclerosis (MS) [28–30].

We recently began to examine the relevance of the endogenous neuropeptides and the role of receptor subtypes by gene deletion in the murine model of experimental autoimmune encephalomyelitis (EAE), a widely used model for MS [31]. MS, the most common neurodegenerative condition in young adults, is a chronic and inflammatory demyelinating disease of the central nervous system (CNS) [32]. Despite its complex pathogenesis, considerable evidence suggests that autoimmune cells reacting against myelin peptides play a major role in the initiation and development of the disease. In fact, EAE is induced by immunizing mice with myelin peptides in order to generate encephalitogenic T cells in the proximal lymph nodes. Both IFNγ-producing Th1 and IL-17-producing Th17 pro-inflammatory cells have been implicated in the pathogenesis of EAE. These T cells migrate into the CNS where, once reactivated, they initiate an inflammatory cascade. We have previously found that PACAP- or VPAC2-targeted gene deletions in mice lead to exacerbated disease, supporting the anti-inflammatory properties of PACAP and VPAC2 [33]. However, we unexpectedly found that VIP knockout (KO) mice developed ameliorated EAE and reduced inflammation in models of LPS-induced endotoxemia and chemically-induced colitis [34–36]. A critical question to be addressed is which VIP receptor mediates this paradoxical resistance to inflammation. Here, we have examined the relevance of VPAC1 in autoimmune CNS inflammation by subjecting VPAC1-deficient mice to EAE.

## Methods

### Mice

Eight to 12-week-old female C57BL/6 (wild-type (WT)) and VPAC1 KO mice [37] were used. Mice were maintained in specific pathogen-free barrier facilities at the University of California, Los Angeles (UCLA). All husbandry and experimental procedures were performed in compliance with the USDA Animal Welfare Act regulations section 2.31(d)(5), Institutional Policies and Guidelines, and adhered to all principles stated in the *Guide for the Care and Use of Laboratory Animals*. The experimental protocol was approved by the UCLA Animal Resource Committee (ARC# 1993-302). VPAC1 KO mice were fed gruel every day starting after weaning on postnatal day 35 to avoid intestinal dysfunction.

### EAE induction

EAE was induced as previously described [38]. Briefly, mice were lightly anesthetized with isoflurane, and a solution containing 100 μg of MOG_35–55_ (GLBiochem) in complete Freund’s adjuvant (Difco) supplemented with 5 mg/ml *Mycobacterium tuberculosis* H37Ra (Difco) was injected subcutaneously in the flanks. Two hundred nanograms of Pertussis toxin (List Biological Laboratories) was administered intraperitoneally (i.p.) to the mice on the immunization day as well as 2 days later. For VPAC1 antagonist pretreatment studies, WT mice were given i.p. either PBS or 10 nmol of the VPAC1 antagonist PG97-269 [39] daily for 2 weeks. Two days after the last dose of the VPAC1 antagonist, EAE was induced as above. For antagonist/agonist treatment studies during ongoing EAE, the VPAC1 antagonist PG97-269 at 10 nmol per mouse or the VPAC1 agonist (Ala^11, 22, 28^)VIP at 5 nmol per mouse were administered i.p. for five consecutive days starting on day 3 after mice immunization with MOG. EAE symptoms were scored daily on a 0–4 scale as follows: 0, no symptoms; 1, wobbling gait; 2, hind leg paralysis; 3, paralysis of two limbs; and 4, moribund or dead.

### Histopathology

The spinal cords were collected 30 days after EAE induction, fixed in 4 % PFA overnight and then gradually dehydrated in ethanol until paraffin embedding. Six-micrometer sections were prepared with a microtome and stained with luxol fast blue (for myelin) and hematoxylin-eosin (for stroma and immune infiltrates) following standard protocols. Histopathology was scored as follows: 0, normal appearance of tissue; 1, scarce immune cell infiltration and demyelination; 2, perivascular infiltrates with a few areas of demyelination; and 3, increasing severity of perivascular cuffing with extension into adjacent tissue and large areas of demyelination.

### Immunofluorescence

Fourteen days after EAE induction, mice were perfused with 4 % PFA and the spinal cords were collected, postfixed overnight, and cryoprotected with a 20 % sucrose solution. Cryostat 15-μm sections were prepared and incubated with anti-CD4 (BD Pharmingen) and anti-laminin (Sigma-Aldrich) antibodies in PBS/1 % bovine serum albumin (BSA)/0.3 % Triton X-100 at 4 °C overnight. Then, sections were incubated for 40 min at room temperature with Alexa 594- and FITC-conjugated secondary antibodies. Slides were mounted using VECTASHIELD with DAPI (Vector Labs).

### Real-time RT-PCR

The brain and spinal cords were collected on day 5, at the clinical peak (days 14–15) or 30 days post-EAE induction and mechanically homogenized in Trizol (Sigma-Aldrich). RNA was extracted according to the manufacturer’s instructions. Before cDNA synthesis, RNA samples were treated with DNase I (DNA-free kit, Ambion) according to the manufacturer’s protocol. One micrograms of total RNA was reverse-transcribed using the iScript kit from Bio-Rad (Hercules). Quantitative real-time RT-PCR for the cytokines tumor necrosis factor (TNF)α, IFNγ, IL-4, IL-6, IL-10, IL-17, and IL-23p19; the chemokines regulated on activation, normal T cell expressed and secreted (RANTES)/CCL5, monocyte chemoattractant protein (MCP)-1/CCL2, MCP-2/CCL8, monokine induced by gamma interferon (MIG)/CXCL9, interferon gamma-induced protein 10 (IP-10)/CXCL10, and MIP-3α/CCL20; the chemokine receptors, CCR1, CCR2, and CCR5; the adhesion molecules intercellular adhesion molecule (ICAM) and vascular cell adhesion molecule (VCAM); and the transcription factor Foxp3 was performed using the iQ SYBR Green Supermix from Bio-Rad. Primers and real-time PCR conditions are described in the Additional file 1: Table S1. For relative quantification, the relative expression was determined by the 2^−ΔΔCt^ method (Ct denoting the threshold cycle of PCR amplification at which product is first detected by fluorescence), which compares the amount of target gene amplification, normalized to the hypoxanthine-guanine phosphoribosyltransferase (HPRT) endogenous reference [40]. Melting curves established the purity of the amplified band. For each probe set, PCR products were sequenced to confirm identity.

### Ex vivo studies

Axillary lymph nodes were collected 14 days after EAE induction and cell suspensions prepared by tapping the organs through a 40-μm nylon mesh. Cells were cultured at 1 × 10^6^ cells/ml in 96-well plates in complete RPMI 1640 (25 mM HEPES, 2 mM l-glutamine, 1 % penicillin/streptomycin, and 2 % FBS), in the presence of MOG or ovalbumin (OVA) at 10 μg/ml. For measurement of cytokines (IFNγ, IL-17A, IL-10), supernatants were collected 48 h later and stored at −20 °C until analysis by ELISA, using antibody kits from PeproTech and eBioscience following manufacturer’s instructions. Because the levels of IL-4 protein were below the detection threshold by ELISA, we determined the expression of this cytokine by real-time RT-PCR. Thus, cells were also collected for RNA extraction with Trizol after the 48-h re-stimulation period. In order to assess cell proliferation, [^3^H]-thymidine (1 μCi/well) was added after 2 days of culture for 18 additional hours, and incorporated radioactivity was measured on a β-scintillation counter (Beckman).

### Adoptive transfer

The lymph node and spleen cells were collected from EAE-induced WT and VPAC1 KO mice on day 12 and restimulated in vitro at 1.5 × 10^6^ or 3 × 10^6^ cells/ml, respectively, in complete RPMI with 30 μg/mL of MOG_35–55_. Three days later, cells (5 × 10^6^ splenocytes and 5 × 10^6^ lymph node cells) were intravenuosly (i.v.) injected into naïve WT or VPAC1 KO recipients. Mice also received 200 ng of pertussis toxin i.p. on days 0 and 2, and clinical symptoms of EAE were scored on a daily basis.

### Bone marrow chimeras

C57BL/6 WT mice were γ-irradiated at 4–6 weeks of age with 550 rads twice with a gap of 2 h to deplete BM immune hematopoietic elements. Following irradiation, mice were provided antibiotics (neomycin sulfate 2 mg/ml) for 2 weeks. Four hours after irradiation, 10^7^ BM cells from either VPAC1 KO (CD45.2) or WT (CD45.2) mice (isolated by flushing the femur with a needle and syringe) were intravenously infused to irradiated mice (CD45.1). The percent donor composition was determined by measuring the percentage of CD45.2 (donor) vs. CD45.1 (recipient) in whole blood cells in the recipient mice before EAE induction. Mice were maintained in quarantine for 6 weeks before EAE induction as above.

### Statistical analysis

Differences between groups were evaluated using GraphPad Prism 6 software (GraphPad Software, San Diego, CA) and Student’s *t* test with Welch’s correction. The level of significance was set at *P* < 0.05. Results are expressed as mean ± SEM.

## Results

### VPAC1 KO mice exhibit reduced clinical and histopathological features of EAE

**Fig. 1:**
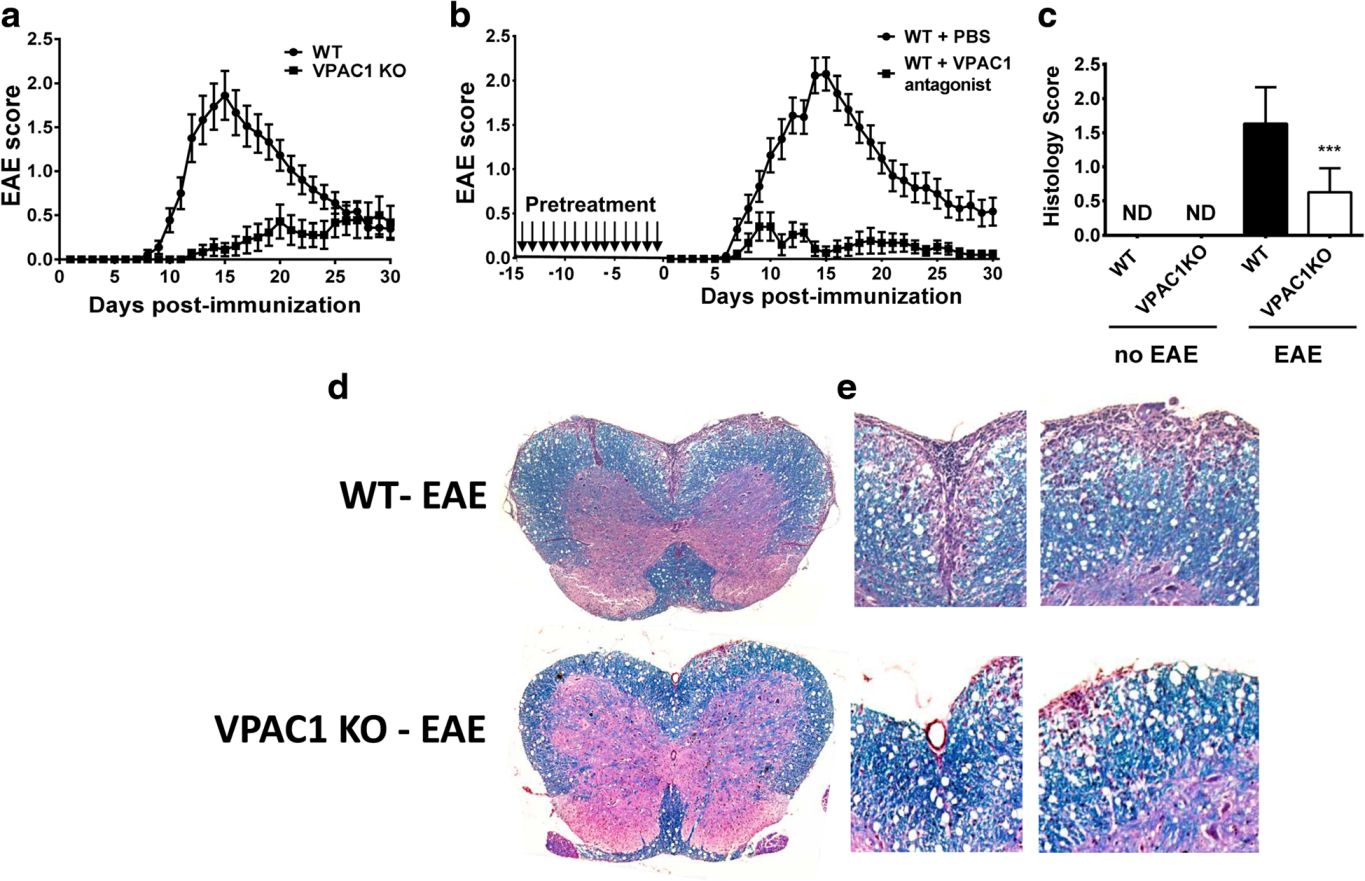
Reduced clinical EAE and histopathology in VPAC1 KO vs. WT mice. EAE was induced by immunizing mice subcutaneously with 100 μg of MOG_35–55_ in CFA supplemented with *Mycobacterium tuberculosis*. EAE clinical scores were monitored daily on a scale of 0 to 4 as described in the “Methods” section. Spinal cord microsections were stained with luxol fast blue (for demyelination) and hematoxylin-eosin (for immune cell infiltration), and histopathology was scored from 0 to 3 as described in the “Methods” section. **a** Clinical curve displaying mean clinical scores ± SEM of immunized WT vs. VPAC1 KO mice, representing the summation of three experiments (*n* = 18 mice per genotype). **b** Clinical curve displaying mean clinical scores ± SEM of immunized WT mice pretreated with PBS or a VPAC1 antagonist, representing the summation of two experiments (*n* = 15 for WT-PBS and *n* = 11 for WT-VPAC1 antagonist). **c** Mean histopathological score at day 30 of three experiments ± SEM (*n* = 12 in each group). ***p < 0.001 Student’s *t* test. ND = not determined. **d** Low- (×4) and **e** high- (×20) magnification photomicrographs of day 30 WT and VPAC1 KO mice transverse thoracolumbar spinal cord sections

In order to investigate the role of the VPAC1 receptor in the development of EAE, we immunized WT and VPAC1 KO mice with MOG_35–55_ and monitored the appearance of clinical symptoms of the disease on a daily basis. We found that EAE clinical symptoms were significantly ameliorated in VPAC1 KO mice. A curve representing the summation of three experiments is shown on Fig. 1a. Indeed, they exhibited lower average cumulative scores (11.2 ± 2.7 vs 25.5 ± 3.4, **p < 0.01) compared to WT mice (calculated as the average sum of all EAE clinical scores in a diseased mouse). In addition, the onset of the disease was delayed in those VPAC1 KO mice who developed EAE (KO average onset of 19.7 ± 1.5 vs 10.5 ± 0.4 days in WT mice, ***p < 0.001). Overall, in the total of three experiments, the incidence of the disease was 50 % for VPAC1 KO mice (9 mice out of 18 developed EAE symptoms) vs. 84 % for WT mice (15 out of 18). To mimic the effect of genetic loss of VPAC1, WT mice were treated with the VPAC1 receptor-specific antagonist (PG97-269 [22]) for 2 weeks prior to EAE immunization. This pretreatment led to a reduced clinical EAE similar to VPAC1 KO mice, although no delay in the onset of the disease was observed (Fig. 1b). These data suggest a critical requirement of the VPAC1 receptor for the development of the full disease. Interestingly, treatment of WT mice with a VPAC1 antagonist for 5 days starting on day 3 post-EAE induction did not block EAE development (Additional file 2: Figure S1), suggesting that either the absence of VPAC1 signaling before disease induction or a long-term blockade of signaling through this receptor is necessary to impede the full development of EAE. Moreover, a short-term treatment with the VPAC1 agonist starting on day 3 post-immunization prevented the development of EAE (Additional file 2: Figure S1), in agreement with the anti-inflammatory activities of VIP.

**Fig. 2:**
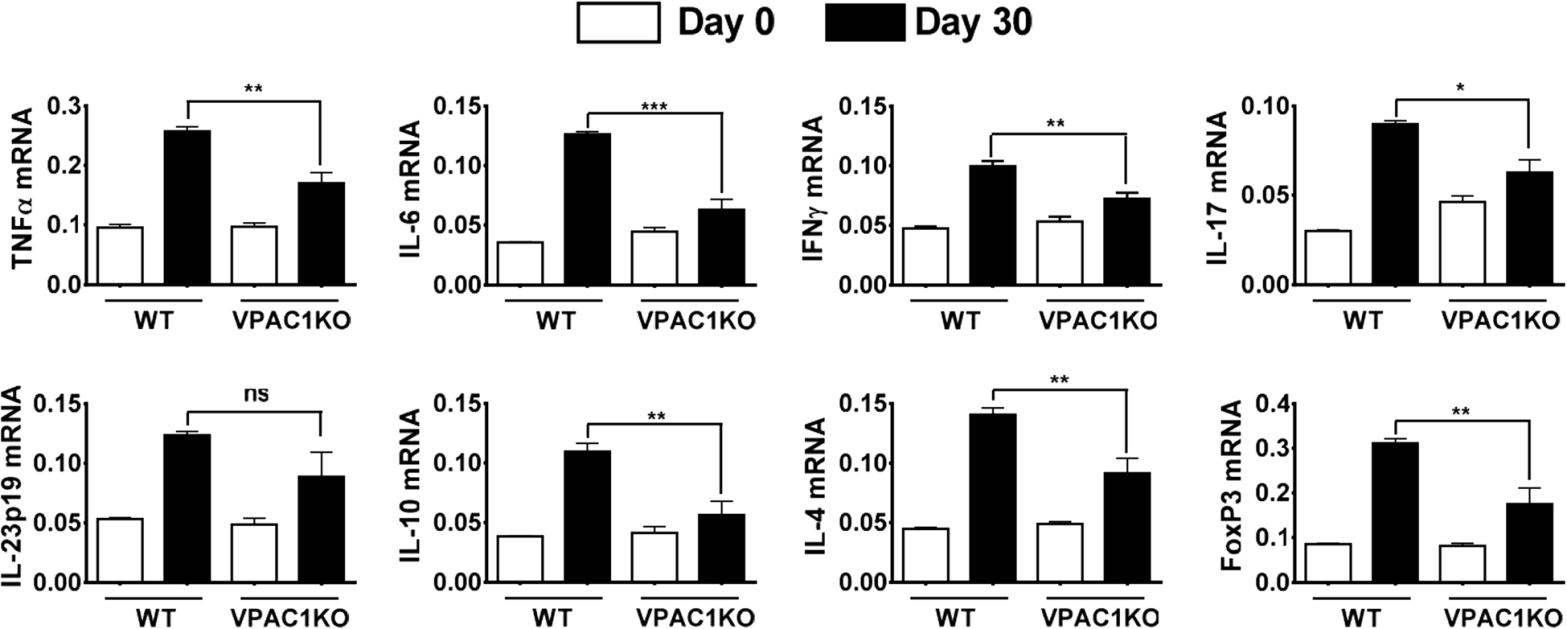
Cytokine expression in the CNS of EAE-immunized mice is reduced in VPAC1 KO mice. EAE was induced in WT- and VPAC1-deficient mice, and the spinal cords were collected and fresh-frozen in liquid nitrogen 30 days later. RNA was extracted and retrotranscribed to cDNA, and the levels of expression of TNFα, IL-6, IFNγ, IL-17, IL-23p19, IL-4, IL-10, and Foxp3 determined by real-time RT-PCR as described in the “Methods” sections. Results shown are representative of three independent experiments of *n* = 8 mice/group. *p < 0.05, **p < 0.01, ***p < 0.001, ns = not significant; Student’s *t* test

In accordance with the reduced clinical disease, the spinal cords of VPAC1 KO mice exhibited minor immune cell infiltration as well as reduced demyelination compared to WT mice 30 days after the induction of the disease, as reflected in the average histological scores (0.6 ± 0.1 in VPAC1 KO vs 1.6 ± 0.1 in WT mice, ***p < 0.001, Fig. 1c) and representative micrographs (Fig. 1d, e). Furthermore, corroborating their lower degree of inflammation in the CNS, we found a generalized lower expression of pro-inflammatory and anti-inflammatory cytokines in the CNS of VPAC1 KO vs. WT mice (TNFα, IL-6, IFNγ, IL-17, IL23p19, IL-4, and IL-10, Fig. 2), as determined on day 30 by real-time PCR. A reduction in the mRNA expression of Foxp3, a transcription factor that specifically marks Tregs, was also observed in the spinal cords of the KO mice. These results show a limited clinical response of VPAC1 KO mice to EAE induction, associated with a reduced immunological response in the CNS.

### VPAC1 KO mice exhibit robust Th1 and Th17 cell responses against MOG despite the lack of clinical EAE

**Fig. 3:**
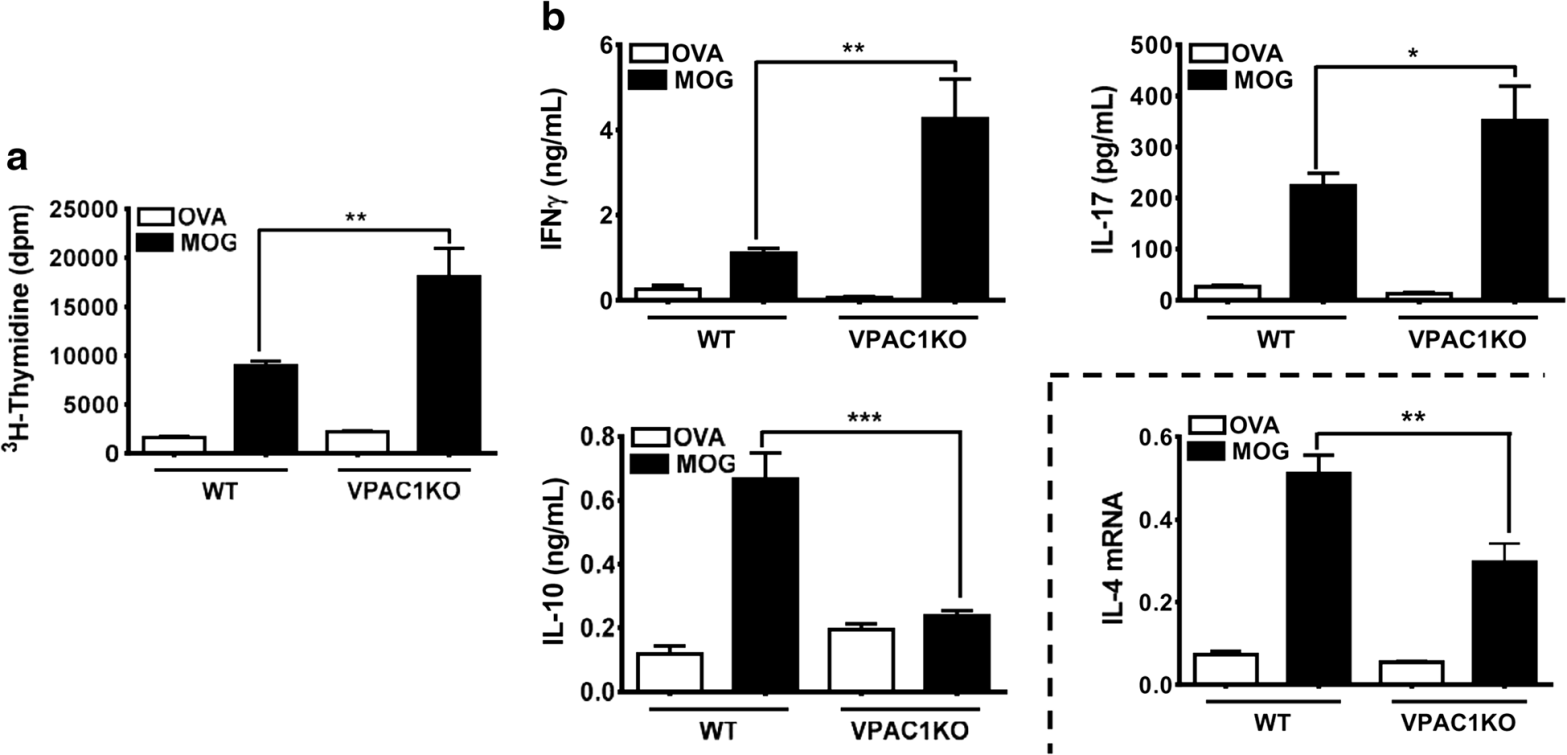
Antigen-recall assay demonstrates robust responses to MOG of lymph node cells from either WT or VPAC1 KO mice after EAE immunization. EAE was induced to WT and VPAC1 KO mice and draining lymph nodes were isolated at the peak of the WT disease (day 14). Cells were cultured for 48 h at 1 × 10^6^ cells/ml in 96-well plates in the presence of OVA or MOG (10 μg/ml). **a** Proliferation determined by measurement of [^3^H]-thymidine incorporation. **b** Cytokine responses in WT vs. VPAC1 KO lymph node cultures. The levels of IFNγ (Th1), IL-17 (Th17), and IL-10 (Th2/Treg) were measured in the supernatants by ELISA. The expression of IL-4 mRNA in the cultured cells was determined by real-time RT-PCR. Results shown are representative of three independent experiments of *n* = 8 mice/group. *p < 0.05, **p < 0.01, ***p < 0.001, Student’s *t* test

The lack of EAE in VPAC1-deficient mice could be due to a defect in MOG immunization. To test this hypothesis, we performed antigen-recall assays in which responses to MOG were examined in cultured lymph node cells isolated from WT or VPAC1 KO mice 14 days after EAE induction. We found that both WT- and VPAC1-deficient mice cells exhibited robust proliferation and production of IFNγ (Th1) and IL-17 (Th17) when stimulated with MOG but not with an irrelevant antigen (OVA) (Fig. 3). Moreover, these were significantly stronger in KO than in WT cultures, suggesting exacerbated Th1 and Th17 responses in these mice. Interestingly, the levels of the anti-inflammatory cytokine IL-10 in the supernatants of VPAC1 KO cultures were significantly lower than those in WT cultures, where this cytokine was highly produced in response to MOG. This cytokine has been shown to be produced by Th2 and regulatory T cells. In addition, the levels of IL-4 (a Th2 cytokine) in our culture supernatants were not detectable by ELISA, but we found a significantly diminished expression of IL-4 mRNA in the MOG-stimulated lymphoid cells of immunized VPAC1 KO mice compared to WT. In summary, the lack of VPAC1 leads to an altered Th response, with enhanced Th1 and Th17 but reduced Th2 profiles, and possibly a diminution of Treg cells. In addition, the ability of Th cells from immunized VPAC1 KO mice to respond to MOG indicate that the mice do not exhibit a defect in the immunization phase of EAE and that the resistance in the mutant mice is not likely due to a compensatory suppression of immunization secondary to VPAC1 loss.

### Impaired immune cell infiltration in the CNS of VPAC1 KO mice

**Fig. 4:**
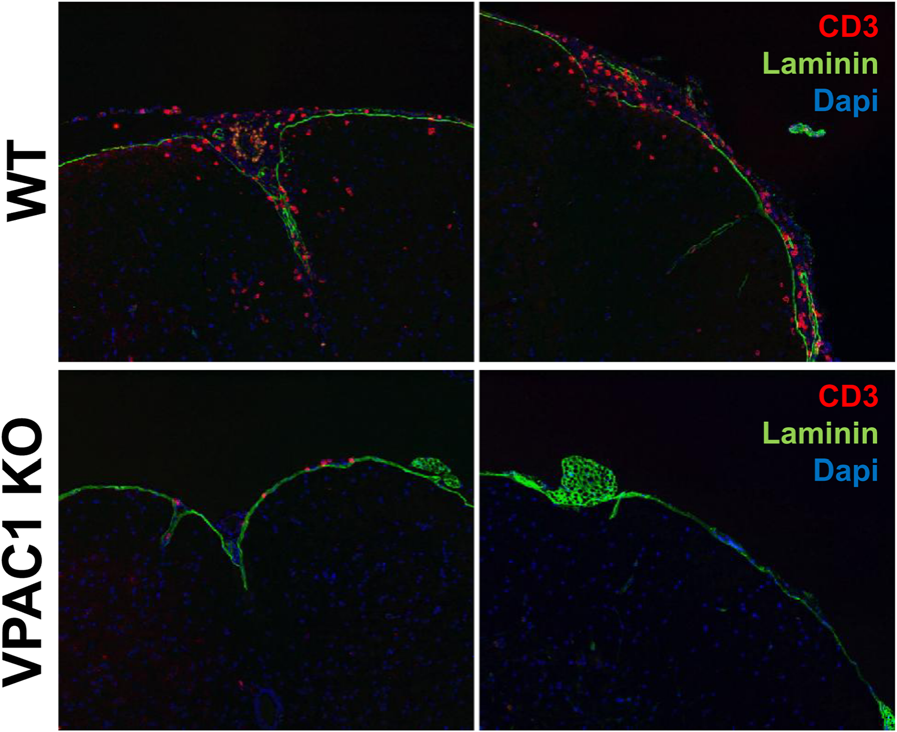
VPAC1-deficient mice exhibit reduced immune cell infiltration into the CNS meninges and parenchyma after EAE induction. EAE was induced in WT and VPAC1 KO mice as described in the “Methods”, and the spinal cord tissues were obtained on day 14 post-immunization. Cryostat section were stained by immunofluorescence for CD4 (Alexa 594), laminin (FITC), and nuclei (DAPI). Representative photomicrographs at ×20 magnification are shown. Results shown are representative of two independent experiments of *n* = 3 mice/group

During the earliest phases of the pathogenesis of EAE, small numbers of encephalitogenic cells enter the subarachnoid space, where it has been suggested that they are reactivated by local antigen-presenting cells [41]. They then cross the blood-brain barrier (BBB) and infiltrate the CNS parenchyma where they initiate an inflammatory response characterized by the recruitment of increasing numbers of immune cells. Our data suggest that VPAC1 KO mice are capable of developing highly reactive lymphocytes in response to EAE immunization. However, these cells may exhibit defects in their ability to migrate into the CNS or fail to amplify the inflammatory response. Thus, in addition to our histopathological studies performed 30 days post-immunization, we determined by immunofluorescence the presence of lymphocytes in the spinal cords of VPAC1 KO vs. WT mice at a time corresponding to the peak of inflammation in WT mice (14 days after immunization) (Fig. 4). We found that, at this time point, significant numbers of T cells had accumulated in the meninges and infiltrated the CNS parenchyma of the WT mice. However, there was no lymphocyte infiltration in the CNS parenchyma of VPAC1 KO-immunized mice. Moreover, there were few/absent lymphocytes in the perivascular areas and in the meninges. These data suggest that VPAC1 KO mice may exhibit a deficit in T cell homing to both the CNS perivascular/meningeal areas and to subsequent CNS parenchyma invasion, a phenotype similar to that we observed in VIP KO mice [17].

### Absence of VPAC1 receptors in either T cells or bone marrow-derived hematopoietic cells does not account for the lack of full EAE development in the VPAC1 KO mice

**Fig. 5:**
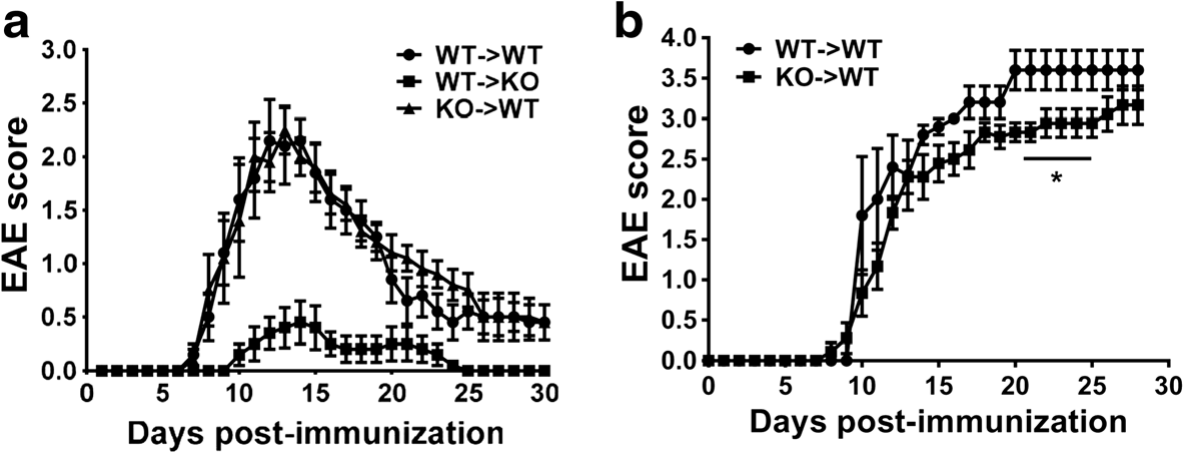
The expression of VPAC1 in the immune/hematopoietic compartment is not critical for the development of full EAE. Adoptive transfer and bone marrow chimera studies were performed in order to investigate the contribution of VPAC1 receptor expression in immune/hematopoietic cells to the resistant phenotype observed in VPAC1 KO mice. **a** For adoptive transfer experiments, 5 × 10^6^ splenocytes and 5 × 10^6^ lymph node cells from EAE-immunized WT and VPAC1 KO mice restimulated with MOG in vitro for 3 days were injected i.v. into naïve WT or VPAC1 KO recipients. The EAE clinical curve of a representative experiment out of three with *n* = 5 for each group is shown. **b** In order to generate bone marrow (BM) chimeric mice, WT mice were γ-irradiated (*n* = 5 for WT→WT and *n* = 9 for VPAC1 KO→WT) and received 10^7^ BM cells from either VPAC1 receptor KO (CD45.2) or WT (CD45.2) mice. Six weeks later, mice were MOG_35–55_-immunized for EAE induction. *p < 0.05, Student’s *t* test

The previous experiments suggest that the ameliorated clinical EAE in VPAC1 KO mice is due to a defect in the effector phase of the disease rather than impaired immunization. To corroborate this possibility, we first performed adoptive transfer studies in which we administered WT or VPAC1 KO immune cells from EAE-immunized mice into WT or VPAC1 KO recipients (Fig. 5a). We found that WT recipients receiving cells from WT donors developed EAE with an onset on day ~8, a peak on days 12–13, and a subsequent recovery phase. These mice developed a similar EAE clinical course when receiving cells from MOG-immunized VPAC1 KO mice, supporting the full encephalitogenic potential of these cells as suggested by antigen-recall assays. In contrast, when VPAC1 KO mice received WT cells, they developed only minor EAE symptoms. Together, the adoptive transfer studies suggest that lymphocytes from the KO mice maintain the potential to induce EAE but that they are not capable of triggering a full inflammatory response in a VPAC1-deficient environment.

The adoptive transfer studies indicated that loss of VPAC1 in encephalitogenic T cells is not sufficient to explain the EAE resistance of VPAC1 KO mice. Peripheral myeloid cells are clearly required to produce full EAE [42, 43], so it remains possible that VPAC1 loss on these or other hematopoietic cells explains the EAE resistance in these mice. To investigate this possibility, we performed bone marrow chimera experiments in which CD45.1 WT mice were γ-irradiated and received CD45.2 WT or CD45.2 VPAC1 KO bone marrow cells. Reconstitution analyses indicated that 85–90 % of immune cells were donors derived in recipients. WT→WT chimeric mice were found to develop chronic EAE with an onset on days 8–9 and no remission of the disease (Fig. 5b). WT chimeric mice with a deletion of VPAC1 in the hematopoietic compartment (KO→WT) also developed EAE in a similar fashion, although the severity of the disease was slightly, although significantly lower between days 20 and 25 than that in WT→WT mice. These results suggest that VPAC1 deficiency in the non-CNS hematopoietic compartment plays a role but is minimally responsible for the remarkable resistance to clinical EAE development found in VPAC1 KO mice.

### The expression of chemokines is reduced in the CNS of MOG-immunized VPAC1 KO vs. WT mice

**Fig. 6:**
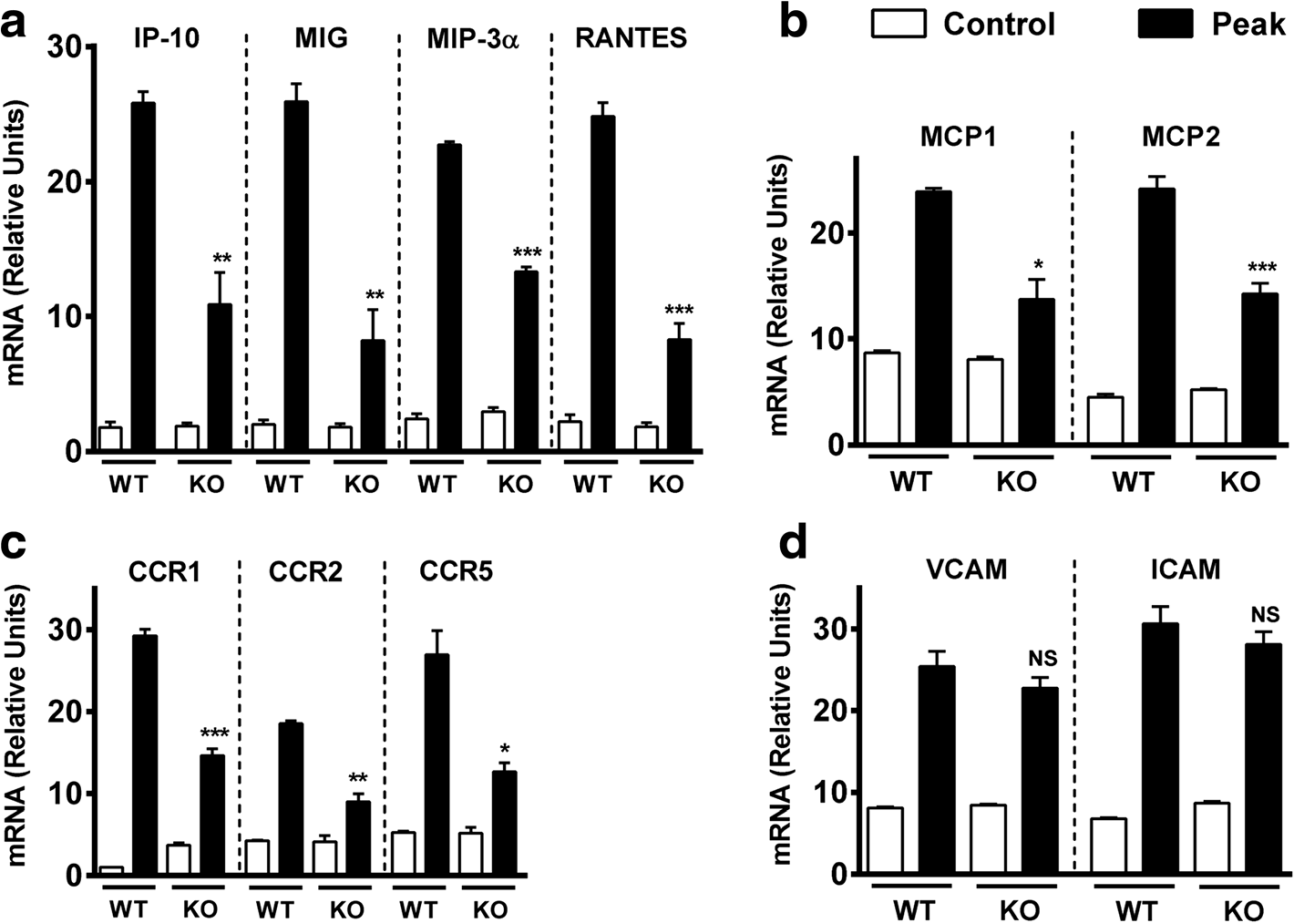
Chemokine and chemokine receptor but not adhesion molecule expressions in the CNS of EAE-immunized mice are reduced in VPAC1 KO mice. EAE was induced in WT- and VPAC1-deficient mice, and the spinal cords were collected and fresh-frozen in liquid nitrogen at the peak of EAE (14–15 days later). RNA was extracted and retrotranscribed to cDNA, and the levels of expression of **a** the lymphocyte chemokines RANTES/CCL5, MIG/CXCL9 (Th1), IP-10/CXCL10 (Th1), and MIP-3α/CCL20 (Th17); **b** the monocyte chemokines MCP-1/CCL2 and MCP-2/CCL8; **c** the chemokine receptors CCR1, CCR2, and CCR5; and **d** the adhesion molecules ICAM and VCAM were determined by real-time RT-PCR as described in the “Methods” sections. Results shown are representative of two independent experiments of *n* = 5 mice/group. *p < 0.05, **p < 0.01, ***p < 0.001, ns = not significant; Student’s *t* test

Our adoptive transfer data suggests that the intrinsic ability of VPAC1 KO immune cells to enter the CNS is not defective. We next investigated by real-time RT-PCR whether or not the expression of chemokines in the spinal cord is impaired in these mice at the peak of EAE (Fig. 6). We found that the expression of RANTES/CCL5, MCP-1/CCL2, and MCP-2/CCL8 and the chemokine receptors CCR1, CCR2, and CCR5 were significantly reduced in VPAC1 KO mice. In addition, we determined the expression of MIG/CXCL9, IP-10/CXCL10, and MIP3-α/CCL20, which are purported to be Th1 (MIG and IP-10)- and Th17-specific (CCL20) chemokines, and found then to be also significantly reduced in VPAC1 KO mice. Similar reductions were found at an earlier time point (day 5 post-immunization), when clinical EAE has not yet manifested (Additional file 3: Figure S2). This could account for the lack of immune infiltration in these mice and suggests a role for VIP/VPAC1 for chemokine induction and cell migration. Moreover, these effects were specific, as we found that the expression of the adhesion molecules ICAM and VCAM was not reduced in VPAC1 KO mice, suggesting that VIP/VPAC1 may not be required for cellular adhesion.

## Discussion

VIP, PACAP, and their receptors have been proposed as potential candidates for the treatment of inflammatory and autoimmune conditions such as MS. Studies utilizing specific VPAC1, VPAC2, and PAC1 agonists have suggested an anti-inflammatory role for all three receptors, although VPAC1 has been implicated as the principle receptor involved [4]. However, our results in peptide and receptor KO mice have brought new information regarding the roles of the endogenous peptides in the pathogenesis of EAE and the receptor subtypes involved. Whereas the neuropeptide PACAP and the VPAC2 receptor appear to prevail as inhibitors of inflammation and clinical disease, VIP and VPAC1 receptors seem to be necessary at some point for EAE development. This new evidence strongly suggests that some aspect of EAE could be potentially blocked pharmacologically with a VPAC1 antagonist. In fact, we report here that a 2-week pretreatment with a VPAC1 antagonist attenuated the development of the disease. This is in contrast with previous studies in the context of multiple models of acute and chronic inflammation (including EAE), in which a short-term treatment with a VPAC1 agonist started at the beginning or during ongoing disease was beneficial [28, 30, 44]. Both the length and the different start points for the treatment might influence dual actions of VIP/VPAC1. In our studies with a VPAC1 antagonist treatment, a long-term administration before EAE induction led to a lack of EAE similar to that found in VPAC1 KO mice. However, a short-term administration during the early phases of EAE did not alter the early EAE course, suggesting that VPAC1 activation during this time frame does not regulate immune cell infiltration. Moreover, in agreement with published studies, we found that a short-term administration of a VPAC1 agonist during early EAE phases blocked EAE development (Additional file 2: Figure S1). The dual stimulatory vs. inhibitory effects of VIP/VPAC1 may depend on different factors such as their level of expression, their target cellular activation status, or their interaction with other mediators present in the microenvironment (i.e., basal vs inflammatory conditions). Variations in the expression of VPAC1 and VPAC2 in immune cells upon exposure to different stimuli have been described. For example, VPAC1 was reported to be constitutively expressed in multiple immune cell types investigated (i.e., T lymphocytes and macrophages) and downregulated after cellular stimulation [45–50]. In contrast, VPAC2 has a low expression in several cellular subtypes that is increased after cellular activation [5, 51]. The consequence of the reciprocal regulation of VPAC1 and VPAC2 in immune cells during inflammation has yet to be determined but could conceivably play a role in the paradoxical EAE resistance of VPAC1 KO and VPAC1 antagonist-pretreated WT mice.

EAE is a multistep pathology in which two phases have been characterized: the immunization and the effector phase. During the immunization phase, the antigen is drained to the lymph nodes located proximal to the injection site and presented by dendritic cells to naïve T cells that in turn become encephalitogenic. Later on, during the effector phase, these cells migrate to the CNS where they trigger an inflammatory response involving the further recruitment of increasing numbers of immune cells as well as the local activation of microglia. The pathogenesis of EAE is considered to be driven by both Th1 and Th17 cells, and their hallmark cytokines, IFNγ and IL-17, are strong activators of the innate arm of immunity. A possible explanation for the lack of EAE development in VPAC1 KO mice would be a requirement for this receptor in the immunization phase of the disease. However, our results show that cells from VPAC1 KO-immunized mice were able to respond to MOG in vitro. Moreover, our results demonstrate stronger Th1 and Th17 but reduced Th2 responses in these mice. These results, which agree with the demonstrated ability of VIP to favor Th2 vs. Th1 responses in vitro and in vivo [16, 18, 21], are similar to those recently found in VPAC2-deficient mice, suggesting an involvement of both receptors on VIP-mediated Th differentiation [33]. Nevertheless, the EAE phenotype in VPAC1 and VPAC2 KO mice is opposite, suggesting that events apart from Th polarization are differentially mediated by VPAC1 and VPAC2. Adoptive transfer is a useful experimental approach as it bypasses the initial phase in which endogenous T cells are exposed to the autoantigen, by injecting cells from immunized mice that have been reactivated in vitro [52]. Upon transfer, lymphocytes from VPAC1 KO mice induced EAE in WT recipients, supporting the normal development of encephalitogenic T cells in these mice upon MOG administration. However, cells from WT mice, which were perfectly capable of inducing the disease in WT recipients, did not transfer EAE to KO mice. This suggests that these cells were not capable of migrating to the CNS, were unable to cross the BBB, or could not be locally reactivated to exert their effector functions. Our immunofluorescence results suggest that the former may be a possibility, since we found very few CD4^+^ cells in VPAC1 KO mice directly immunized with MOG. In an attempt to determine whether or not immune cells are retained in the lymphoid organs or the peripheral circulation of VPAC1 KO mice, we have measured and compared the numbers of white blood cells and percentages of lymphocytes and monocytes in WT vs. VPAC1 KO mice during EAE. We found that the number of lymphocytes in the blood of VPAC1 KO mice on day 9 post-immunization was significantly higher than in WT mice, which might be due to the inability of these cells to enter the brain (Additional file 4: Figure S3). In the lymph nodes, the total number of cells in WT and VPAC1 KO mice before EAE and at the peak of the disease were not significantly different (Additional file 5: Figure S4).

Several in vitro studies have shown a direct effect of VIP on T cell chemotaxis. A study demonstrated a chemoattractant activity for VIP on unstimulated and stimulated T cells, although the effect was less pronounced in the latter [53]. In that study, VIP treatment of T cells augmented their adhesion to ICAM and VCAM in vitro. These molecules play an important role in the adhesion of T cells to the endothelium and their subsequent transmigration. In addition, VIP increased T cell adhesion to fibronectin, a component of the extracellular matrix, through which T cells must migrate upon extravasation. Moreover, VIP increased the production of metalloprotease 9 (MMP-9) by the lymphoblastoma T cell line Tsup1 and increased cell migration through a layer of basement membrane-like Matrigel [54]. In another study, VIP and a VPAC1 ligand stimulated chemotaxis of human T and B lymphocytes into micropore filters [55]. Finally, a transcriptomic analysis of VIP-regulated genes in murine isolated TCD4^+^ splenic lymphocytes revealed an increased expression of multiple genes related to homing and migration upon VIP treatment in vitro concomitant to PMA and ionomycin activation [56]. Despite these findings, VPAC1 KO immune cells induced EAE upon transfer to WT recipients, suggesting that their intrinsic ability to migrate is not impaired, and that the EAE-resistant phenotype is dependent on VPAC1 receptor loss in another cell type. Although it is still possible that the prior ex vivo re-stimulation of lymphocytes artificially restored this function, our bone marrow chimera studies, in which the WT hematopoietic/immune compartment was replaced by that of VPAC1 mice before EAE induction, also support that VPAC1 expression in this compartment has little relevance for the development of full disease. However, it is well known that microglia are not eliminated by irradiation by this bone marrow chimera protocol, so it remains possible that loss of VPAC1 in this hematopoietic cell type explains much of the EAE resistance of VPAC1 KO mice.

Our data suggests that the absence of VPAC1 may disrupt the mechanisms involved in the migration of immune cells into the CNS in EAE. In order for this to occur, cells must cross the BBB, which has several molecular and cellular components such as the tight junctions between the endothelial cells and the astrocytic feet or *glia limitans*. Activation of microglia and secretion of pro-inflammatory mediators, metalloproteases, and chemokines are also necessary in order to promote cell migration and to amplify the inflammatory response. There is evidence suggesting that these cell types express VIP/PACAP receptors [57–60], and acting on them, they could modulate immune cell migration. The absence of VPAC1 in such cells may interfere with the entrance of the first T cells into the CNS or in the subsequent immune cell infiltration during the amplification step. Among all factors involved in cell migration during EAE, Arima et al. highlighted a critical role for IL-6 for the initial entry of immune cells through the lumbar region of the spinal cord [61]. Interestingly, it has been shown that VIP promotes the secretion of IL-6 by resting macrophages [62], and we found this cytokine among others to be downregulated in MOG-immunized VIP and VPAC1 KO mice. Both Arima et al. and Reboldi et al. [63] have suggested that CCL20 is necessary in the first immune cell entry into the CNS, and we also found this chemokine to be reduced in these mice. Interestingly, the expression of all cytokines and chemokines we measured was decreased in VPAC1 KO mice, whereas the expression of adhesion molecules was similarly induced in WT and VPAC1 KO mice. The latter suggest that the defects are specific for the chemokine system. Whether or not VIP is required for transmigration of immune cells by modulating the physical architecture or functioning of the BBB remains to be elucidated.

One of the properties of VIP that might be related to cell migration is its ability to elicit vasodilation. Other vasodilatory peptides, such as adrenomedullin, which causes vasodilation in the cerebral circulation, have been suggested to play an important role in the regulation of specific BBB properties as endothelium-derived autocrine/paracrine hormones [64, 65]. Vasodilation is likely to increase BBB permeability [66]. The vasodilatory actions of VIP and PACAP in brain arteries have been demonstrated in the context of migraine. Dilatation of the intracranial vasculature is considered a putative trigger factor for the activation of nociceptors, which has been involved in the pathogenesis of migraine. It has been shown that administration of VIP or PACAP causes headache in healthy volunteers, albeit with significantly less potency of VIP compared to PACAP [67, 68]. Nevertheless, whether or not this general vasodilatory activity plays a role in immune cell migration across the BBB remains to be elucidated.

Another report has also described a reduced response of VPAC1 KO mice to inflammation. In this study, VPAC1 KO mice developed a milder colitis induced with the chemical dextran sodium sulfate (DSS) than WT mice [69]. In that report, the authors suggested that VIP through VPAC1 signaling may exhibit pro-inflammatory actions through the induction of Th17 responses or mediating immune cell migration to the gut. The resistance of VPAC1 KO mice to inflammatory insult in the DSS colitis and EAE models is reminiscent of that found in mice deficient in VIP (the ligand) in EAE and other models of inflammation such as TNBS-induced colitis and endotoxemia induced by a lethal dose of LPS, a model of acute inflammation with no Th1/Th17 implication [34–36]. We have proposed that the reduced inflammation in VIP KO mice is due, at least partially, to certain defects in the innate immune response, because peritoneal cells from these mice produced reduced levels of the pro-inflammatory cytokines TNFα and IL-6 in response to LPS [35]. Both stimulatory and inhibitory actions for VIP on these cytokines have been reported in different experimental settings [62, 70, 71]. Nevertheless, whether or not this is the case in VPAC1-deficient mice remains to be elucidated. Despite the fact that VIP-VPAC1 could potentially play an active role to promote immune responses, other possibilities to explain the resistance of the KO mice for these molecules, such as compensatory anti-inflammatory mechanisms, cannot be excluded at this time. Interestingly, this has been shown to be the case in mice deficient in cortistatin [72]. Despite the anti-inflammatory actions of this neuropeptide, cortistatin-deficient mice were paradoxically resistant to EAE development, which was found to be associated to basal elevated circulating glucocorticoids and an anxiety-like behavior. Conversely, we have not found increased serum corticosterone levels in basal conditions or in response to an acute stressor in VIP KO mice, although this remains to be elucidated in the context of EAE [73].

**Fig. 7:**
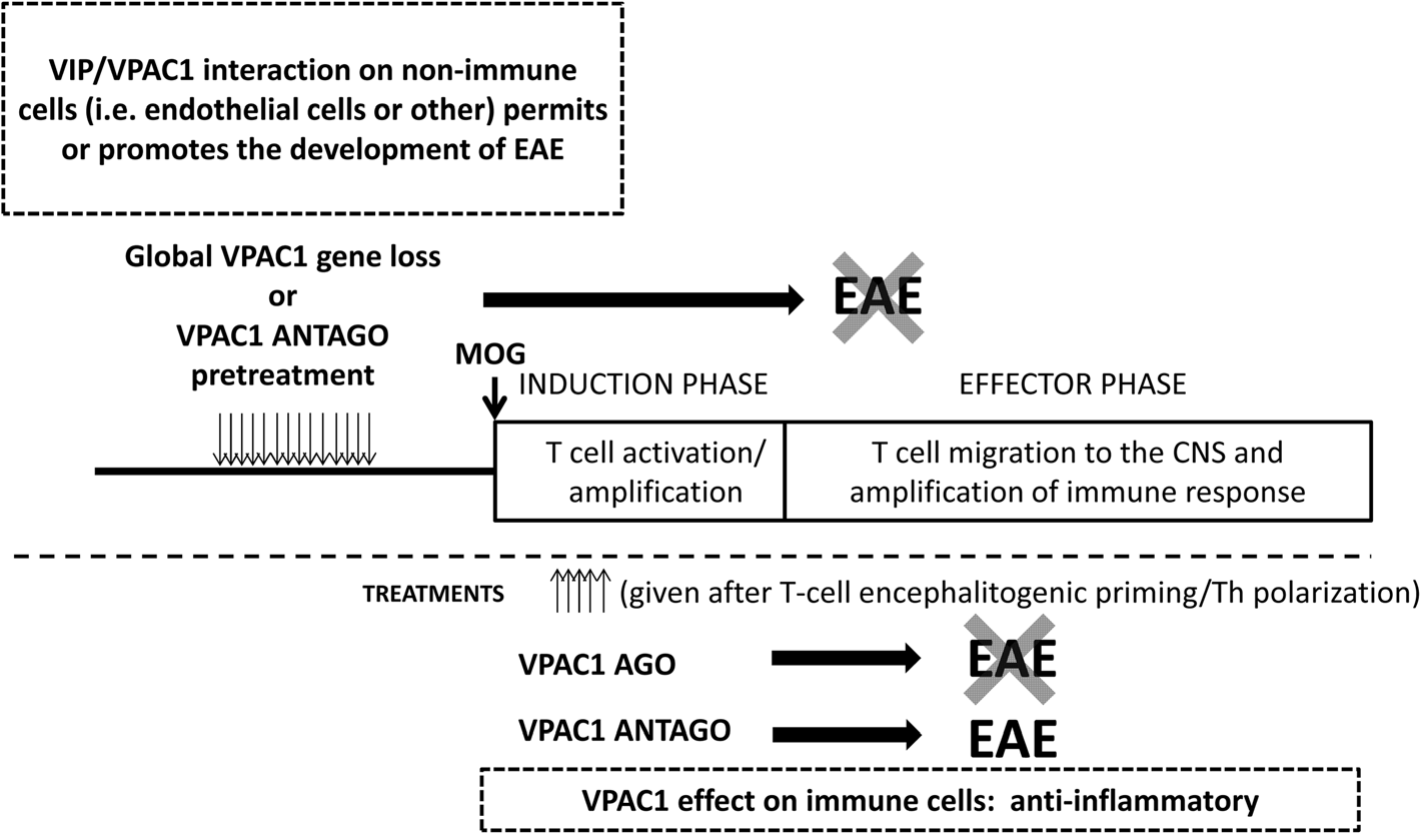
Scheme suggesting potential roles of VPAC1 on EAE. Upon MOG administration, EAE has two phases: the induction or priming phase and the effector phase. In the induction phase, MOG drains to the local lymph nodes and T cells are activated and polarized towards Th1 and Th17 cells. In the effector phase, these cells migrate to the CNS, where they mount an inflammatory response amplified by immune cell recruitment. Our data suggests that VPAC1 signaling on non-immune cells or microglia may be required for the effector phase of EAE and may explain the lack of clinical disease in VPAC1 KO mice or upon pretreatment of WT mice with a VPAC1 antagonist (VPAC1 antago). VPAC1 anti-inflammatory actions would be prevalent only during ongoing disease, and thus a VPAC1 agonist (VPAC1 ago) administered during this time blocked the disease. A treatment with a VPAC1 antagonist during ongoing disease during the same time frame post-EAE induction did not prevent and may have exacerbated the disease. Mice deficient in one of the two VPAC1 ligands, VIP, are also resistant to EAE, whereas PACAP-deficient mice exhibit exacerbated EAE, implying a critical VIP/VPAC1 interaction that is required for the development of EAE

Our prior results using KO mice have shown that VPAC2 receptor and PACAP KO mice developed exacerbated EAE as expected, supporting the primarily anti-inflammatory properties of these endogenous molecules in untreated mice. Data from pharmacological studies show that major anti-inflammatory effects of exogenously administered VIP and PACAP in EAE models are mediated by VPAC1, although a VPAC2 receptor-specific agonist was shown to be also anti-inflammatory. Conversely, VIP and VPAC1 KO mice were resistant to full EAE development. This unexpected phenotype in VPAC1 receptor-deficient mice suggests a specific requirement of VIP/VPAC1 interaction required for the full development of autoimmune inflammation (Fig. 7). While our studies so far have shown that the administration of a VPAC1 agonist during the initial stages of EAE development suppressed EAE, it remains to be determined if chronic administration of a VPAC1 agonist would begin to worsen EAE, as might be suggested by the EAE resistance of mice with chronic global loss of VPAC1.

## Conclusions

In this study, we have found that the VIP/PACAP receptor VPAC1 is required for the full development of EAE, as VPAC1 KO mice exhibited an unexpectedly but strikingly ameliorated clinical course of the disease. The facts that (1) in vitro*,* antigen-rechallenged lymph node cells from VPAC1 KO mice respond strongly to MOG*,* (2) VPAC1 KO lymph node cells induce EAE upon transfer to WT mice, and (3) bone marrow chimeric VPAC1 KO→WT mice develop full EAE suggest that the deficient ability of VPAC1 KO mice to develop EAE is not due to impairments in the immunization phase of the disease. Our study suggests that VPAC1 may play different roles in the pathogenesis of EAE, i.e., permissive in the early stages vs. anti-inflammatory if pharmacologically targeted during ongoing inflammation (Fig. 7). Thus, the use of agonists vs. antagonists of VPAC1 should be carefully examined to achieve maximum beneficial effects in diseases such as MS.

## Abbreviations

BBB, blood-brain barrier; BSA, bovine serum albumin; CNS, central nervous system; EAE, experimental autoimmune encephalomyelitis; HPRT, hypoxanthine-guanine phosphoribosyltransferase; ICAM, intercellular adhesion molecule; IFN, interferon; IL, interleukin; IP-10, interferon gamma-induced protein 10; IUPHAR, International Union of Basic and Clinical Pharmacology; LPS, lipopolysaccharide; MCP, monocyte chemoattractant protein; MIG, monokine induced by gamma interferon; MIP, macrophage inflammatory protein; MMP, metalloprotease; MOG_35–55_, myelin oligodendrocyte glycoprotein 35–55; MS, multiple sclerosis; OVA, ovalbumin; PACAP, pituitary adenylate cyclase-activating polypeptide; PCR, polymerase chain reaction; PFA, paraformaldehyde; RANTES, regulated on activation, normal T cell expressed and secreted; Th, T helper; TNF, tumor necrosis factor; Treg, regulatory T cell; VCAM, vascular cell adhesion molecule; VIP, vasoactive intestinal peptide; WT, wild-type

## Additional files

Additional file 1: Table S1. Oligonucleotide primers used for real-time PCR (PDF 236 kb) Additional file 2: Figure S1. Effects of a VPAC1 agonist or antagonist treatment started on early EAE. EAE was induced by immunizing mice subcutaneously with 100 μg of MOG_35–55_ in CFA supplemented with *Mycobacterium tuberculosis*, and EAE clinical scores were monitored daily on a scale of 0 to 4 as described in the “Methods” section. The clinical curve displays the mean clinical scores ± SEM of immunized WT mice treated with PBS, the VPAC1 antagonist PG97-269 at 10 nmol per mouse, or the VPAC1 agonist (Ala^11, 22, 28^)VIP at 5 nmol per mouse for five consecutive days starting on day 3 (indicated by the arrow). A representative experiment out of two is shown (*n* = 10 for each group). (PDF 14 kb) Additional file 3: Figure S2. Early chemokine expression in the CNS of EAE-immunized mice is reduced in VPAC1 KO mice. EAE was induced in WT and VPAC1-deficient mice, and the spinal cords were collected and fresh-frozen in liquid nitrogen on day 5. RNA was extracted and retrotranscribed to cDNA and the levels of expression of IP-10, MIG, MIP-3α, and RANTES determined by real-time RT-PCR as described in the “Methods” sections. Results shown are representative of two independent experiments of *n* = 8 mice/group,***p* < 0.01,****p* < 0.001, ns = not significant; Student’s *t* test. (PDF 18 kb) Additional file 4: Figure S3. Quantification of white blood cell populations in WT and VPAC1 KO mice. EAE was induced to VPAC1 KO and WT mice and retro-orbital blood collected at different time points (days 6, 9, and 15). Cells were counted using a XT-4000i haematology analyser from Sysmex. *n* = 4 per group. (PDF 4 kb) Additional file 5: Figure S4. The total numbers of cells in the draining lymph nodes of WT and VPAC1 KO mice do not differ. EAE was induced in WT and VPAC1-deficient mice, and the draining lymph nodes isolated at the peak of the disease. Lymph nodes from naïve animals served as controls. A cell suspension was prepared by tapping the organs through a 40-μm nylon mesh, and cells counted with a hemocytometer (*n* = 5 for each group). (PDF 8 kb)
