# Erratum to: VPAC1 receptor (Vipr1)-deficient mice exhibit ameliorated experimental autoimmune encephalomyelitis, with specific deficits in the effector stage

**DOI:** 10.1186/s12974-017-0927-1

**Published:** 2017-08-04

**Authors:** Catalina Abad, Bhavaani Jayaram, Laurine Becquet, Yuqi Wang, M. Sue O’Dorisio, James A. Waschek, Yossan-Var Tan

**Affiliations:** 1 Department of Psychiatry, David Geffen School of Medicine, University of California, Los Angeles, USA; 2 Inserm U905, Institute for Research and Innovation in Biomedicine (IRIB), University of Rouen, Normandy, France; 3 Department of Pediatrics and Holden Comprehensive Cancer Center, RJ and LA Carver College of Medicine, University of Iowa, Iowa City, IA 52242 USA

## Erratum

After publication of the article [1], it has been brought to our attention that one of the authors has had their name spelt incorrectly. The fourth author should be “Yuqi Wang”. This has been updated in the original version of the article.
