# Supplementary material for: VPAC1 receptor (Vipr1)-deficient mice exhibit ameliorated experimental autoimmune encephalomyelitis, with specific deficits in the effector stage
Source: J Neuroinflammation. 2016 Jun 29;13:169. doi: 10.1186/s12974-016-0626-3 (PMC4928347; doi:10.1186/s12974-016-0626-3)
Supplement: Additional file 1: Table S1. — Oligonucleotide primers used for real-time PCR (PDF 236 kb) [file 12974_2016_626_MOESM1_ESM.pdf]

Supplementary table 1. Oligonucleotide primers used for Real time PCR

Gene	GenBank Accession Number	Primer Sequence	
		Sense	Antisense
**Chemokines**			
**IP-10/CXCL10**	NM_021274	5′-TGCTGGGTCTGAGTGGGACT-3′	5′-CCCTATGGCCCTCATTCTCAC-3′
**MCP-1/CCL2**	NM_011333	5’-AAAAACCTGGATCGGAACCAA-3’	5’-CGGGTCAACTTCACATTCAAAG-3’
**MCP-2 /CCL8**	NM_021443	5’-GGGTGCTGAAAAGCTACGAG-3’	5’-TCCAGCTTTGGCTGTCTCTT-3’
**MIG/CXCL9**	NM_008599	5′-TCTGCCATGAAGTCCGCTG-3’	5′-CAGGAGCATCGTGCATTCCT-3’
**MIP-3α/CCL20**	NM_016960	5′-CAAGCGTCTGCTCTTCCTTG-3’	5-TGGATCAGCGCACACAGATT-3’
**RANTES/CCL5**	NM_013653	5’-ACACCACTCCCTGCTGCTTT-3’	5’-GACTGCAAGATTGGAGCACTTG-3’
**Chemokine receptors**			
**CCR1**	NM_009912	5-CCATCCACAGGAGAACATG-3’	5-GCCCCATTTTGTTTATTCA-3’
**CCR2**	NM_009915	5-AAAGGAAATGGAAGACAATAATATG-3’	5-AAAGGCAAACTGTCACTTACTTTAC-3’
**CCR5**	NM_009917	5-TGGCTTCTCCACACAAACAA-3’	5-CGGAGCTTGAGAAAAAACCAG-3’
**Adhesion molecules**			
**ICAM-1**	NM_010493	5’-CATCCCAGAGAAGCCTTCCTG-3’	5’-TCAGCCACTGAGTCTCCAAGC-3’
**VCAM-1**	NM_011693	5’-AACGACCTTCATCCCCACC-3’	5'-TCTGCCTCTGTTTGGGTTCAG-3’3’
**Cytokines**			
**IFNγ**	NM_008337	5’-TGCTGATGGGAGGAGATGTCT-3’	5’-TTTCTTTCAGGGACAGCCTGTT-3’
**IL-4**	NM_021283	5’-CGAGGTCACAGGAGAAGGGA-3’	5’-AAGCCCTACAGACGAGCTCACT-3’
**IL-6**	NM_010551	5’-TTCCATCCAGTTGCCTTCTTG-3’	5’-TTGGGAGTGGTATCCTCTGTGA-3’
**IL-10**	NM_010548	5’-GGTTGCCAAGCCTTATCGGA-3’	5’-ACCTGCTCCACTGCCTTGCT-3’
**IL-17A**	NM_010552	5’-GAAGCTCAGTGCCGCCA-3’	5’-TTCATGTGGTGGTCCAGCTTT-3’
**IL-23p19**	NM_031252	5’-CCAGCAGCTCTCTCGGAATC-3’	5’-TCATATGTCCCGCTGGTGC-3’
**TNFα**	NM_013693	5’-CGATCACCCCGAAGTTCAGTA-3’	5’-GGTGCCTATGTCTCAGCCTCTT-3’
**Transcription factors**			
**Foxp3**	NM_054039	5’-GGCCCTTCTCCAGGACAGA-3’	5’-CTGATCATGGCTGGGTTGT-3’
**Housekeeping Gene**			
**HPRT**	NM_013556	5’-TGGTGAAAAGGACCTCTCGAA-3’	5’-TCAAGGGCATATCCAACAACA-3’

The amplification protocols used were: initial denaturation at 96°C for 5 min for all and 40 cycles of: 96°C for 25 secs followed by 60°C for 35 secs, and 72°C for 35 secs for IFNγ, IL-4, MCP-1, ICAM, VCAM, CCR1, CCR5; 96°C for 20 secs, 60°C for 30 secs, and 72°C for 20 secs for IL-6, IL-10, IL-17A, IL-23p19, RANTES and MIP-3α; 96°C for 20 secs, 62°C for 30 secs, and 72°C for 20 secs for TNFα, MIG and IP-10 and 95°C for 1 min, 60°C for 1 min and 72°C for 1 min for Foxp3 and CCR2, with a final elongation at 72°C for 10 min for all.

Supplementary Figure legends

Supplementary Figure 1. Effects of a VPAC1 agonist or antagonist treatment started on early EAE. EAE was induced by immunizing mice subcutaneously with 100 μg of $MOG_{35\text{-}55}$ in CFA supplemented with *Mycobacterium tuberculosis,* and EAE clinical scores were monitored daily on a scale of 0 to 4 as described in the *Methods* section. The clinical curve displays the mean clinical scores ± SEM of immunized WT mice treated with PBS, the VPAC1 antagonist PG97-269 at 10 nmoles per mouse or the VPAC1 agonist $(Ala^{11, 22, 28})$VIP at 5 nmoles per mouse for five consecutive days starting on day 3 (indicated by the arrow). A representative experiment out of two is shown (n=10 for each group).

Supplementary Figure 2. Early chemokine expression in the CNS of EAE immunized mice is reduced in VPAC1 KO mice. EAE was induced in WT and VPAC1 deficient mice and spinal cords were collected and fresh-frozen in liquid nitrogen on day 5. RNA was extracted and retrotranscribed to cDNA, and the levels of expression of IP-10, MIG, MIP-3α and RANTES determined by real time RT-PCR as described in the *Methods* sections. Results shown are representative of two independent experiments of *n* = 8 mice/group.,**p<0.01,***p<0.001, ns=not significant; Student's *t*-test.

Supplementary Figure 3. Quantification of white blood cell populations in WT and VPAC1 KO mice. EAE was induced to VPAC1 KO and WT mice, and retro-orbital blood collected at different time points (days 6, 9 and 15). Cells were counted using a xs-800i haematology analyser from Sysmex. N=4 per group.

Supplementary Figure 4. The total numbers of cells in the draining lymph nodes of WT and VPAC1 KO mice do not differ. EAE was induced in WT and VPAC1 deficient mice, and the draining lymph nodes isolated at the peak of the disease. Lymph nodes from naïve animals served as controls. A cell suspension was prepared by tapping the organs through a 40 μm nylon mesh, and cells counted with a hemocytometer (n=5 for each group).
