# Supplementary figures and images for: VPAC1 receptor (Vipr1)-deficient mice exhibit ameliorated experimental autoimmune encephalomyelitis, with specific deficits in the effector stage
Source: J Neuroinflammation. 2016 Jun 29;13:169. doi: 10.1186/s12974-016-0626-3 (PMC4928347; doi:10.1186/s12974-016-0626-3)

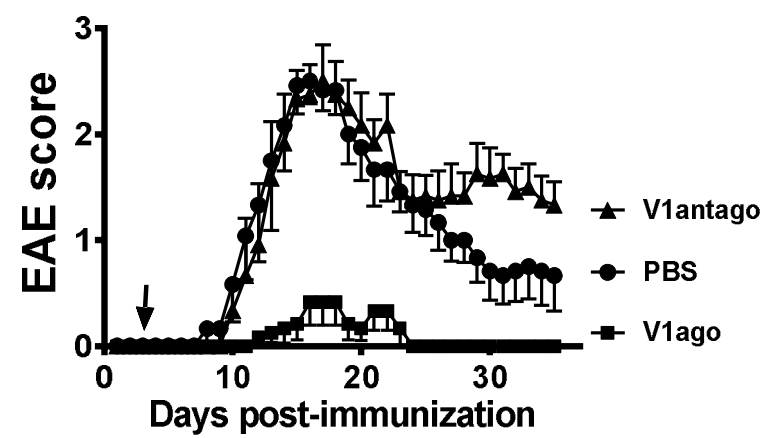

Supplement: Additional file 2: Figure S1. — Effects of a VPAC1 agonist or antagonist treatment started on early EAE. EAE was induced by immunizing mice subcutaneously with 100 μg of MOG35–55 in CFA supplemented with Mycobacterium tuberculosis, and EAE clinical scores were monitored daily on a scale of 0 to 4 as described in the “Methods” section. The clinical curve displays the mean clinical scores ± SEM of immunized WT mice treated with PBS, the VPAC1 antagonist PG97-269 at 10 nmol per mouse, or the VPAC1 agonist (Ala11, 22, 28)VIP at 5 nmol per mouse for five consecutive days starting on day 3 (indicated by the arrow). A representative experiment out of two is shown (n = 10 for each group). (PDF 14 kb) [file 12974_2016_626_MOESM2_ESM.pdf]

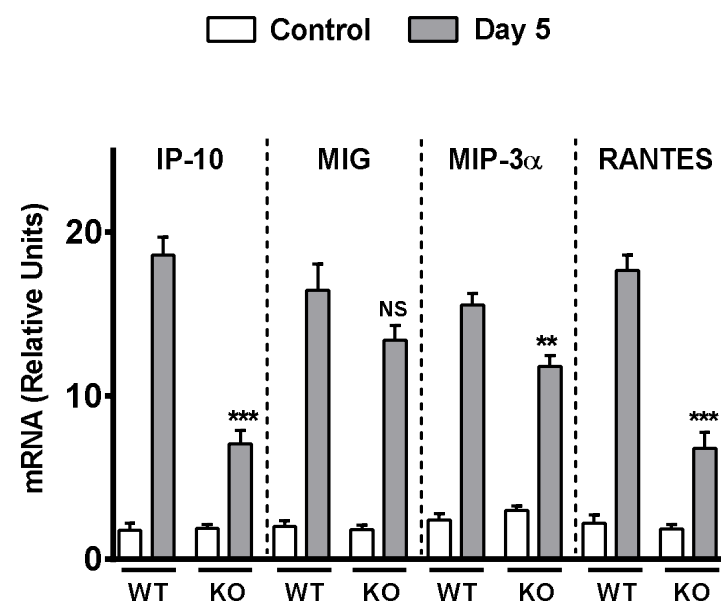

Supplement: Additional file 3: Figure S2. — Early chemokine expression in the CNS of EAE-immunized mice is reduced in VPAC1 KO mice. EAE was induced in WT and VPAC1-deficient mice, and the spinal cords were collected and fresh-frozen in liquid nitrogen on day 5. RNA was extracted and retrotranscribed to cDNA and the levels of expression of IP-10, MIG, MIP-3α, and RANTES determined by real-time RT-PCR as described in the “Methods” sections. Results shown are representative of two independent experiments of n = 8 mice/group,**p < 0.01,***p < 0.001, ns = not significant; Student’s t test. (PDF 18 kb) [file 12974_2016_626_MOESM3_ESM.pdf]

■ WT      □ VPAC1KO

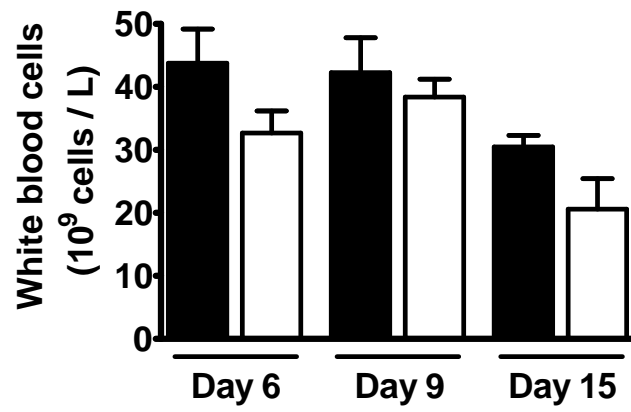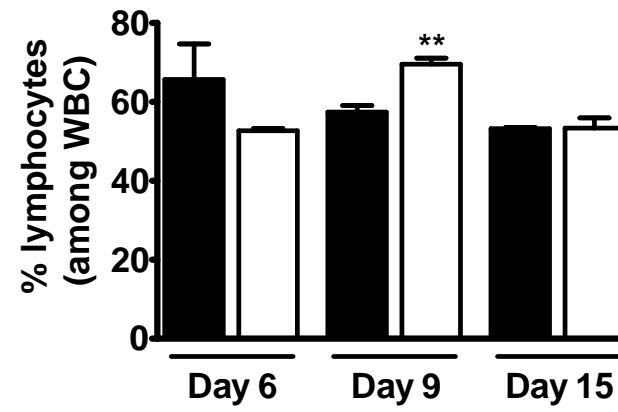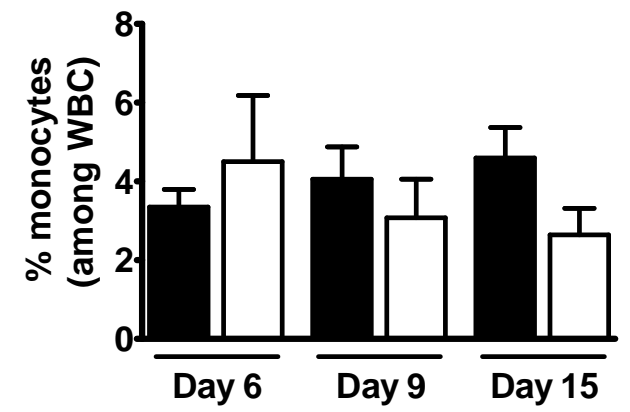

Supplement: Additional file 4: Figure S3. — Quantification of white blood cell populations in WT and VPAC1 KO mice. EAE was induced to VPAC1 KO and WT mice and retro-orbital blood collected at different time points (days 6, 9, and 15). Cells were counted using a XT-4000i haematology analyser from Sysmex. n = 4 per group. (PDF 4 kb) [file 12974_2016_626_MOESM4_ESM.pdf]

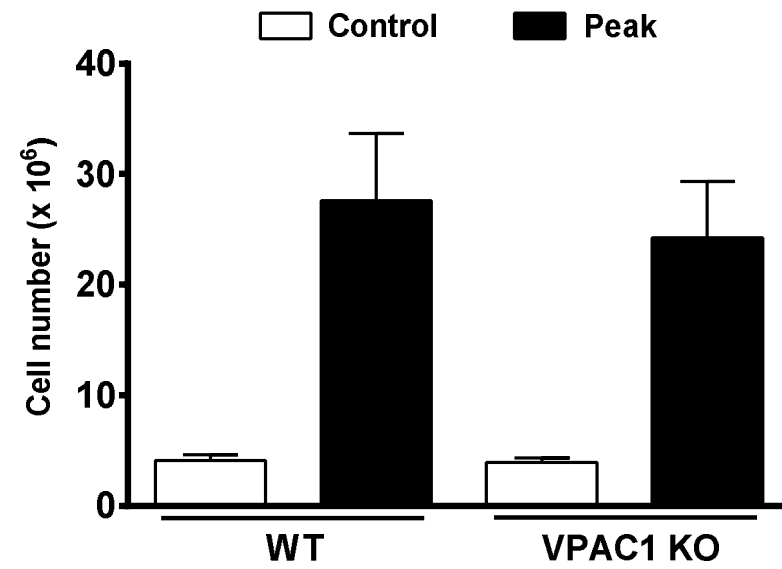

Supplement: Additional file 5: Figure S4. — The total numbers of cells in the draining lymph nodes of WT and VPAC1 KO mice do not differ. EAE was induced in WT and VPAC1-deficient mice, and the draining lymph nodes isolated at the peak of the disease. Lymph nodes from naïve animals served as controls. A cell suspension was prepared by tapping the organs through a 40-μm nylon mesh, and cells counted with a hemocytometer (n = 5 for each group). (PDF 8 kb) [file 12974_2016_626_MOESM5_ESM.pdf]
